# Oral treatment for diabetes using α-glucosidase inhibitors was a risk factor for chronic obstructive pulmonary disease: a cohort study

**DOI:** 10.7150/ijms.55361

**Published:** 2021-01-01

**Authors:** Sheng-Wen Wu, Yung-Chyuan Ho, Ci-Wen Luo, Hung-Yi Chen, Chun-Hung Su, Yu-Hsiang Kuan

**Affiliations:** 1 Division of Nephrology, Department of Internal Medicine, Chung Shan Medical University Hospital, Taichung, Taiwan; 2 The School of Medicine, Chung Shan Medical University, Taichung, Taiwan; 3 School of Medical Applied Chemistry, Chung Shan Medical University, Taichung, Taiwan.; 4 Department of Pharmacology, School of Medicine, Chung Shan Medical University, Taichung, Taiwan; 5 Department of Pharmacy, Chung Shan Medical University Hospital, Taichung, Taiwan; 6 School of Pharmacy, China Medical University, Taichung, Taiwan.; 7 Department of Internal Medicine, Chung Shan Medical University Hospital, Taichung, Taiwan; 8 Department of Internal Medicine, School of Medicine, Chung Shan Medical University, Taichung, Taiwan; 9 Institute of Medicine, Chung Shan Medical University, Taichung, Taiwan

**Keywords:** diabetes mellitus, chronic obstructive pulmonary disease, α-glucosidase inhibitor, cohort study, cox regression

## Abstract

**Objectives:** Currently, diabetes mellitus (DM) and chronic obstructive pulmonary disease (COPD) have proven to be risk factors for each other. This study aimed to determine the risk relationship between COPD and five common oral medications for DM among patients with DM.

**Methods:** This population-based cohort study was conducted from 2008 to 2013. Patient data were retrieved from the Longitudinal Health Insurance Database (LHID) of the National Health Insurance Research Database (NHIRD). After pairing by gender, age, and index date, time-to-event analysis and multiple regression analysis were performed to determine the factors associated with COPD in patients taking oral medication for DM, including age, gender, income, and comorbidities. We identified 1,028 patients who took oral medication for DM and 1,028 controls who did not take oral medication for DM.

**Results:** We observed that the use of α-glucosidase inhibitors was associated with a higher risk of COPD (hazard ratio [HR]: 1.964, 95% confidence interval [CI]: 1.207-2.380). Furthermore, compared with the control group, α-glucosidase inhibitor users had a higher risk of COPD (HR: 2.295, 95% CI: 1.304-4.038), and no significant difference was observed in other oral medications for DM.

**Conclusions:** Based on present results, we could suggest that patients with DM who used α-glucosidase inhibitors are probably a higher risk of COPD. We recommend that in the future, treatment with α-glucosidase inhibitors upregulate the occurrence of COPD might through gastrointestinal side effects and malnutrition.

## Introduction

The relationship between diabetes mellitus (DM) and chronic obstructive pulmonary disease (COPD) has currently been confirmed.^1^,^2^ The literature has also reported that the risk of death in patients with COPD and DM is higher than that in patients with general COPD.^3^ Several medicines have yielded satisfactory results in the treatment of COPD. Potassium channel modulators can effectively dilate the bronchi, reduce cough and mucus production, and inhibit tracheal inflammation^4^. A corresponding study was also conducted on oral medicines for DM. The use of metformin was not related to the deterioration of COPD, but the concentration of plasma lactic acid slightly increased in patients in a statistically significant manner.^5^ Studies have also demonstrated that metformin can reduce mortality in patients with DM and in those with COPD and DM.^3^

Most reports investigated the effects of oral medicines for DM on patients with COPD. We studied the effect of oral medicines for DM on the subsequent development of COPD in patients. In the future, patients with DM should have more options and receive additional recommendations in the use of medicines. This study used the Taiwan National Health Insurance Research Database (NHIRD) to determine the relationship between oral medicines for DM and the development of COPD in patients after a series of adjustments.

## Methods

### Data source

The NHIRD is a claims database covering 98% of the population in Taiwan. The data are derived from the National Health Insurance programme implemented by the National Health Research Institutes. The Taiwan Longitudinal Health Insurance Database 2010 (LHID 2010) consists of a sample of 1 million claims randomly drawn from the NHIRD. No significant difference was observed in the statistical age, gender, annual births, and average insured amount. The strength of this database for research purposes hinges on its large sample size and vertical nature.^6^ It provides information on patient characteristics, medical service, hospital drug compensation, general practice, community pharmacy, and International Classification of Diseases, Ninth Revision, Clinical Modification (ICD-9-CM) diagnostic codes. All NHIRD applicants must be researchers or clinicians in universities, research institutions, or hospitals. The data form NHIRD must be used for research purposes only. All applications should be reviewed by peer experts to ensure the rationality of the research. Ethical approval for this research was obtained from the institutional review board (IRB) of Chung Shan Medical University (CS2-15106).

### Participants

Patients with DM were identified according to the ICD-9-CM code 250, A181. The identification of patients with COPD was conducted using ICD-9-CM codes 490.xx-496.xx. A patient with DM or COPD with at least one hospital admission or three or more outpatient claims was considered to be newly diagnosed as having DM or COPD. The index date was the date of DM diagnosis between 2008 and 2013, and the end point was the date of COPD diagnosis. Exclusion criteria were as follows: (1) missing data including gender and residential area, (2) patients younger than 20, (3) end point before the index date, (4) patients diagnosed as having DM or COPD before 2008, (5) and less than 90 days between the index date and the end point. The patients who used oral medication were defined as cases. We randomly selected individuals not using oral medicine, and we gender-matched, age-matched, and index date-matched them with the patients from the case group to form the control group. There were 1,028 and 1,028 controls.

### Oral medicines for DM

Based on the Anatomical Therapeutic Chemical classification system, we divided patients between biguanides, α-glucosidase inhibitors, sulphonylureas, meglitinides, thiazolidinediones (TZDs), and no oral medicine use for DM. We excluded patients who used more than one medication or were treated with insulin during the follow-up period. Patients having taken medicine for at least a month were included in the study.

### Comorbidity

COPD-related comorbidity^7^ included hypertension (ICD-9-CM code: 401.xx, 402.xx, 403.xx, 404.xx, 405.xx), hyperlipidemia (ICD-9-CM code: 272.xx), cerebrovascular disease (CVD; ICD-9-CM code: 430.xx, 431.xx, 432.xx, 433.xx, 434.xx, 435.xx, 436.xx, 437.xx, 438.xx), anxiety (ICD-9-CM code: 300.0), substance abuse (ICD-9-CM code: 304.xx, 305.xx), congestive heart failure (ICD-9-CM code: 428.xx), peripheral vascular disease (ICD-9-CM code: 443.9), depression (ICD-9-CM code: 311.xx), gastro-esophageal refluxdisease (GERD; ICD-9-CM code: 530.81).

### Statistical analysis

The chi-square test was used to analyse the category variables between the case and control groups. A two-tailed test was used to compare the continuous variables. Univariate and multivariate stratified Cox regression models were subsequently used to calculate the hazard ratio (HR) and 95% CI. Multivariable models were adjusted for COPD-related comorbidities, gender, age, low income, and urbanisation level.^8^ A further analysis of the risk relationship between users of oral medicine for DM and their controls was conducted. Statistical analyses were performed using the SAS 9.3 software package, and P < 0.05 was considered statistically significant.

## Results

From 1 January 2008 to 31 December 2013, a total of 1,028 oral medicine users were compared with 1,028 controls. The descriptive demographic data namely age, gender, income, urbanization level, and comorbidities are presented in Table 1. Patients taking oral medicine were compared with their controls. No difference existed in age and gender. Patients were mostly male (55.88%) and 57 years old on average. There were more patients with a low income in the case group (50.58%) and more patients with a non-low income in the control group (50.88%). No difference was observed between the case and control groups. The patients generally lived in moderately urbanized areas (cases: 29.67%, controls: 30.64%).

Table 2 presents the Cox regression analysis of risk factors associated with COPD development. The HR of α-glucosidase inhibitor users was 1.697 (95% CI: 1.208-2.383) and was statistically significant (P = 0.0023). Low income was also a risk factor for COPD (HR: 1.143, 95% CI: 1.044-1.25, P = 0.0037). Compared with patients living in moderately urbanized areas, those living in agricultural areas had a higher risk of developing COPD (HR: 1.482, 95% CI: 1.197-1.835, P = 0.0003). Patients with hyperlipidaemia (HR: 0.808, 95% CI: 0.738-0.884, P < 0.0001) and cardiovascular disease (HR: 0.874, 95% CI: 0.771-0.991, P = 0.0352) had a lower risk of COPD development.

**Figure 2:**
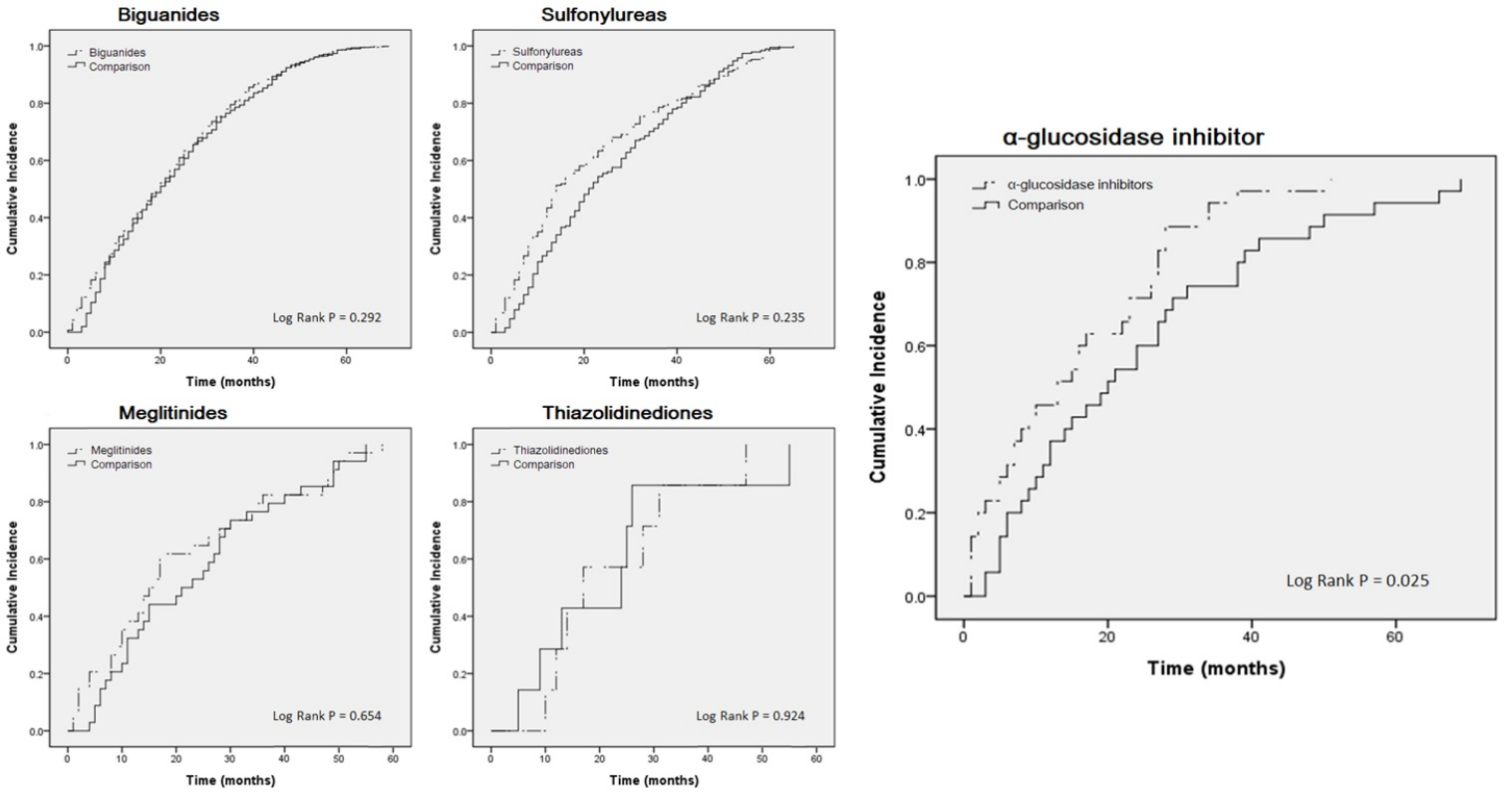
Kaplan-Meier curves estimating cumulative incidence of COPD patients between oral treatment medicine users and control cohorts.

For further analysis, we compared the case group to the control group. Table 3 indicates that, apart from the α-glucosidase inhibitor users (crude HR: 1.718, 95% CI: 1.052-2.807; adjusted HR: 2.295, 95% CI: 1.304-4.038), no statistically significant differences were observed among the users of other medicines. A Kaplan-Meier curve was also used for analysis (Fig. 2). Only α-glucosidase inhibitor users had a significantly higher incidence of COPD than did the controls (log-rank test, P = 0.025).

## Discussion

In this study, patients with DM using α-glucosidase inhibitors had a higher risk of developing COPD than those using other oral medicines. Sulphonylureas bind to and shut down the ATP-sensitive potassium channel on the cell membrane of the pancreatic beta cell, and they prevent the potassium from depolarising by blocking it.^9^ In turn, the fusion of insulin particles with the cell membrane increases, and so does the secretion of mature insulin. The potassium channel has been proven to effectively alleviate the symptoms of COPD, such as decreased airway hyper responsiveness, bronchiectasis, decreased cough, and decreased mucus production as well as inhibition of airway inflammation and remodeling.^10^ Therefore, closing the potassium channel may cause COPD to worsen. One of the sulphonylureas, glyburide, which binds to (+)-**[**^3^H] isradipine, causes pathological changes in the cardiopulmonary structure and function of rats with monocrotaline-induced pulmonary hypertension. This evidence suggests that sulphonylureas had a tendency to aggravate lung injury and related diseases such as COPD. Our results reveal that sulphonylureas were trending towards the development of COPD, but the statistics were nonsignificant. The mechanism of action of meglitinide was the same as that of sulphonylureas, that is, shutting down the ATP-dependent potassium channel.^11^ In contrast to sulphonylureas, meglitinide has a fast onset and a short duration of action. Compared with those caused by sulphonylureas, the side effects of hypoglycaemia and weight gain caused by meglitinide are relatively mild.^12^ This may also affect patients with COPD because of the inhibition of the potassium channels. Our results indicated that patients who used meglitinide did not face a risk of COPD; instead they exhibited a decreasing trend compared with their matched group. Studies have demonstrated that for patients with DM, repaglinide can replace meglitinide and treat early cystic fibrosis-related diseases.^13^ Therefore, it may also be useful in the treatment of COPD. However, the detailed pathological relationship requires clarification.

One of the biguanides, metformin appears to be irrelevant for the treatment of COPD, regardless of whether the patient has diabetes.^14^,^15^ However, studies have demonstrated that metformin can effectively inhibit the mortality of patients with COPD and the development of COPD.^16^, ^17^ Therefore, whether biguanides inhibit the development of COPD remains to be discussed. In our results, there was no significant difference in the risk of COPD between biguanides cases and controls. Patients with COPD and DM who were exposed to TZDs had a small but significant risk of acute exacerbations of COPD.^18^ TZDs exert antidiabetic effects by activating the mechanism of the γ isoform of the peroxisome proliferator-activated receptor (PPARγ) (nuclear receptor) and expression of PPARγ in alveolar macrophages, an in vitro alveolar macrophage model and in vivo associated with COPD Animal model studies have displayed the potential to fight inflammation.^19^ However, studies have also indicated that the long-term use of TZDs in patients with type 2 diabetes causes pneumonia or lower respiratory tract infections as well as severe pneumonia or lower respiratory tract infections. The risk is increased.^20^ TZDs also increases the risk of heart failure (HF)^21^ and HF is often highly correlated with COPD.^22^ In our results, TZDs were not associated with COPD in patients with DM.

The results indicated that patients with DM who used α-glucosidase inhibitors are probably a higher risk of COPD. α-Glucosidase inhibitors, the pseudo-carbohydrates, competitively inhibit activity of α-glucosidases which hydrolyze non-absorbable oligosaccharides and polysaccharides into absorbable monosaccharides in the brush border of enterocytes.^23^ α-Glucosidase inhibitors delay carbohydrate digestion and prolong the carbohydrate digestion duration, thus reducing monosaccharides absorption rates.^24^,^25^ Therefore, patients taken with α-glucosidase inhibitors may have the potential to develop into malnutrition. Addition, α-glucosidase inhibitors cause the gastrointestinal side effects, such as bloating, nausea, diarrhea, and flatulence.^24^ Malnutrition in COPD is described by variable prevalence rates ranging between 30-60%.^26^ Malnutrition and poor nutrition play as the risk factor for patients with COPD. Compared with healthy individuals, patients with COPD had significantly higher rates of 0-3 hours of urinary lactulose to rhamnose and sucralose to erythritol and 5-24 hours of urinary galactooligosaccharide to erythritol.^25^ These findings indicated that intestinal permeability would be significant reduction in carbohydrate metabolism in the patients with COPD^25^ The patients with COPD also suffered by lower gastrointestinal symptoms, including constipation and bloating. The study suggests improving the management of gastrointestinal symptoms and maintaining a clear bowel to improve the condition of patients with COPD.^24^ Based on these findings, the gastrointestinal side effects and malnutrition caused by α-glucosidase inhibitors may be one of the main reasons for the development of COPD, and further experiments need to be clarified. We recommend that patients with DM use α-glucosidase inhibitors in combination with other medicines to alleviate the gastrointestinal side effects and malnutrition.

In the analysis of multiple population studies, poor education systems, low-income families, and low composite socioeconomic status (SES) indices were associated with individuals with COPD whose annual income is below the minimum wage in the United States.^27^ It have a much greater impact on smoking-related diseases, which is the same as ours.^28^ In Poland, 8.5% of men and 4.9% of women have symptoms of chronic airflow obstruction. Livestock farmers have an increased risk of chronic bronchitis, COPD, and reduced forced expiratory volume in 1 second (FEV1).^29^ Exposure to mineral dust by working in the soil has also been suggested to result in COPD.^30^ This was also confirmed in our results. Interestingly, among the patients in the case group, those with DM with high blood lipids or cardiovascular disease had a lower risk of COPD. Studies have demonstrated that patients with hyperlipidaemia and COPD experience lung hyperinflation and airway obstruction less often than patients without hyperlipidaemia, but the effects of the drug need to be clarified.^29^ In fact, α-glucosidase inhibitors can effectively reduce triglycerides and increase HDL; they can also inhibit the risk of cardiovascular disease^32^ Moreover, biguanides and TZDs effectively reduce the risk of cardiovascular disease.^33^,^34^ But, the risk of heart failure is increased by TZDs.^21^ However, the relationship between the risk of cardiovascular disease and anti-diabetic agents including sulphonylureas and meglitinides are not clear.^35^ At present, we also found that there are more patients using biguanides due to the lower risk of cardiovascular disease. Therefore, we further compared various drug users with their controls, but the results were similar.

There are several limitations to this study. We did not have a clear understanding of the lives or exercise habits of patients, for example, their cigarette smoking habit. We also could not accurately obtain the actual values of blood sugar levels, FVC, and FEV1 in patients with diabetes. Although we did not directly track the severity of diabetes and COPD from NHIRD could not directly track patients, adjusted these comorbidities of COPD for indirectly explain the severity. These comorbidities present high-risk factors for COPD. Despite the large sample size, the number of participants for whom a comparison between several medicines could be made remained limited after strict screening and matching. Especially for patients with DM who used TZDs, the result was unexpected. For other users of oral medicine for DM, we believe that there is credible by adjustment and matching.

## Conclusion

In summary, we suggest that patients with DM who administration of the α-glucosidase inhibitors are probably a higher risk of COPD. Although α-glucosidase inhibitors have a satisfactory inhibitory effect on blood lipids and cardiovascular diseases. However, the gastrointestinal side effects and malnutrition of the α-glucosidase inhibitors probably results in higher incidence of COPD occur in the patients with DM. In future medications, the side effects of α-glucosidase inhibitors should be alleviated and the occurrence of COPD reduced.

## Figures and Tables

**Figure 1:**
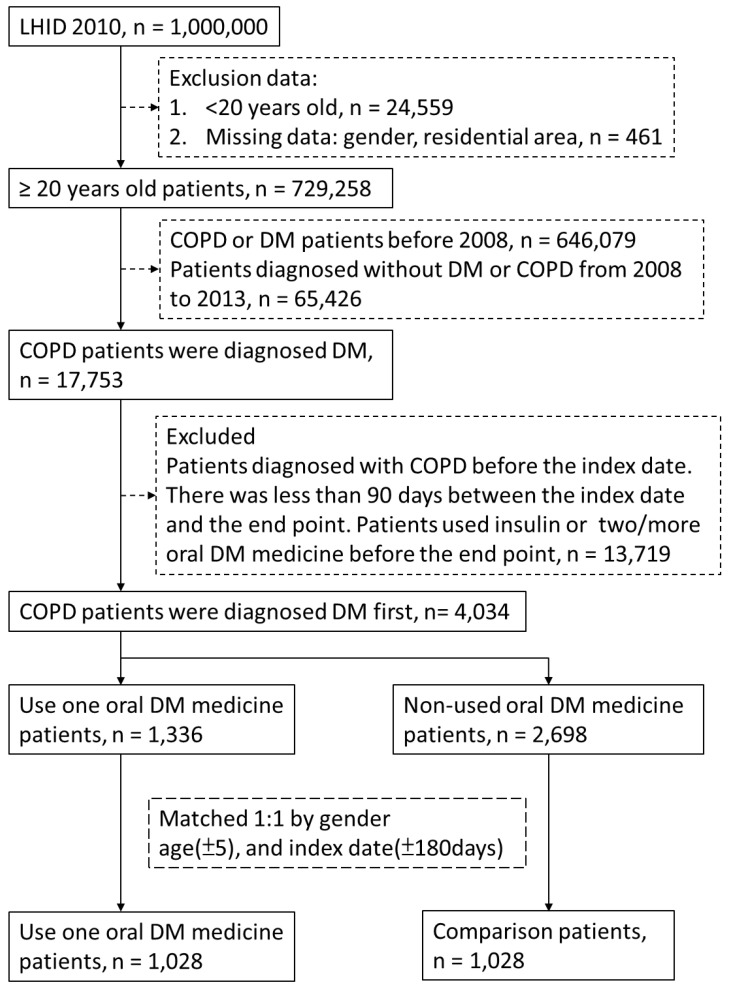
Flow-chart of participant selection.

**Table 1:** Basic characteristics of the study participants from 2008 to 2013.

		Oral medicine			Comparison			
		(n = 1,028)			(n = 1,028)			*P*
**Gender**								
	female	505	(49.12%)		505	(49.12%)		1.0000
	male	523	(50.88%)		523	(50.88%)		
**Age in 2008 (Mean ± SD)**		57.14±13.64			57.23±13.79			0.8773
**Low-income**								
	Yes	505	(49.12%)		520	(50.58%)		0.5082
	No	523	(50.88%)		508	(49.42%)		
**Urbanization level**								
	Highly urbanization	303	(29.47%)		252	(24.51%)		0.0741
	Moderate urbanization	315	(30.64%)		305	(29.67%)		
	Emerging town	156	(15.18%)		167	(16.25%)		
	General town	146	(14.2%)		159	(15.47%)		
	Aged Township	20	(1.95%)		27	(2.63%)		
	Agricultural town	42	(4.09%)		61	(5.93%)		
	Remote township	46	(4.47%)		57	(5.54%)		
**Comorbidity**								
	Hypertension	569	(55.35%)		693	(67.41%)		<.0001
	Hyperlipidemia	514	(50%)		594	(57.78%)		0.0004
	Osteoarthritis	410	(39.88%)		351	(34.14%)		0.0070
	Cardiovascular disease	160	(15.56%)		195	(18.97%)		0.0411
	Anxiety	340	(33.07%)		294	(28.6%)		0.0280
	Substance abuse	32	(3.11%)		30	(2.92%)		0.7965
	Congestive heart failure	54	(5.25%)		64	(6.23%)		0.3430
	Peripheral vascular disease	30	(2.92%)		40	(3.89%)		0.2239
	Depression	31	(3.02%)		27	(2.63%)		0.5942
	GERD	28	(2.72%)		17	(1.65%)		0.0973

Abbreviation: SD, standard deviation.

**Table 2:** Hazards ratios of COPD with diabetes and compare with oral treatment medicine.

		Adjusted		
		HR 95%CI		*P-value*
**Diabetes medicine (reference: no used)**				
	Biguanides (n = 761)	1.089 (0.990-1.198)		0.0812
	α-glucosidase inhibitor (n = 35)	1.694 (1.207-2.380)		0.0023
	Sulfonylureas (n = 191)	1.080 (0.922-1.264)		0.3409
	Meglitinides (n = 34)	1.054 (0.746-1.488)		0.7656
	Thiazolidinediones (n = 7)	1.233 (0.584-2.604)		0.5828
**Gender (reference: female)**				
	Male	1.015 (0.926-1.113)		0.7444
**Age (reference: general population)**				
	Age	0.999 (0.995-1.003)		0.6085
**Low income (reference: no)**				
	Yes	1.143 (1.044-1.250)		0.0036
**Urbanization level (reference: moderate urbanization)**				
	Highly urbanization	1.085 (0.967-1.218)		0.1650
	Emerging town	1.078 (0.941-1.236)		0.2764
	General town	1.080 (0.940-1.241)		0.2755
	Aged Township	0.972 (0.719-1.314)		0.8529
	Agricultural town	1.482 (1.197-1.835)		0.0003
	Remote township	1.041 (0.841-1.288)		0.7138
**Comorbidity (reference: without)**				
	Hypertension	1.027 (0.929-1.135)		0.6052
	Hyperlipidemia	0.808 (0.738-0.884)		<.0001
	Osteoarthritis	0.926 (0.839-1.023)		0.1309
	Cardiovascular disease	0.874 (0.771-0.991)		0.0352
	Anxiety	0.954 (0.864-1.053)		0.3501
	Substance abuse	0.930 (0.716-1.209)		0.5881
	Congestive heart failure	0.977 (0.806-1.184)		0.8122
	Peripheral vascular disease	0.900 (0.706-1.146)		0.3910
	Depression	0.964 (0.737-1.261)		0.7916
	GERD	1.097 (0.812-1.482)		0.5465

Abbreviation: CI, confidence interval. Adjusted with gender, age, low income, urbanization level, comorbidity.

**Table 3:** Oral treatment medicine subgroups of hazards ratios of COPD with diabetes.

		Event	Observed	Incidence Density	Crude		Adjusted	
			Person-months	(Per 1000 person-month)	HR 95%CI	*p*-value	HR 95%CI	*p*-value
**Biguanides medicine**								
	Biguanides	761	16589	45.87	1.054 (0.953-1.166)	0.3056	1.063 (0.960-1.719)	0.2411
	Comparison	761	17512	43.46	Reference		Reference	
**α-glucosidase inhibitor medicine**								
	α-glucosidase inhibitors	35	546	64.10	1.718 (1.052-2.807)	0.0307	2.295 (1.304-4.038)	0.0040
	Comparison	35	837	41.82	Reference		Reference	
**Sulfonylureas medicine**								
	Sulfonylureas	191	4796	39.82	1.127 (0.921-1.379)	0.2471	1.172 (0.946-1.451)	0.1466
	Comparison	191	4074	46.88	Reference		Reference	
**Meglitinides medicine**								
	Meglitinides	34	710	47.89	1.112 (0.688-1.798)	0.6641	0.959 (0.538-1.711)	0.9590
	Comparison	34	808	42.08	Reference		Reference	
**Thiazolidinediones medicine**								
	Thiazolidinediones	7	159	44.03	0.948 (0.314-2.862)	0.9242	-	0.9993
	Comparison	7	157	44.59	Reference		Reference	

Abbreviation: CI, confidence interval. Adjusted with gender, age, low income, urbanization level, comorbidity.
